# Mitochondrial somatic mutations and the lack of viral genomic variation in recurrent respiratory papillomatosis

**DOI:** 10.1038/s41598-019-53148-8

**Published:** 2019-11-12

**Authors:** Yuhan Hao, Ryan Ruiz, Liying Yang, Antonio Galvao Neto, Milan R. Amin, Dervla Kelly, Stratos Achlatis, Scott Roof, Renjie Bing, Kasthuri Kannan, Stuart M. Brown, Zhiheng Pei, Ryan C. Branski

**Affiliations:** 1 Center for Genomics and Systems Biology, Department of Biology, New York University, New York, NY USA; 2 Otolaryngology-Head and Neck Surgery, New York University School of Medicine, New York, NY USA; 3 Department of Pathology, New York University School of Medicine, New York, NY USA; 4 Department of Medicine, New York University School of Medicine, New York, NY USA; 5 Applied Bioinformatics Laboratories, New York University School of Medicine, New York, NY USA; 6 Department of Veterans Affairs New York Harbor Healthcare System, New York, NY USA

**Keywords:** Immunopathogenesis, Infection

## Abstract

Recurrent Respiratory Papillomatosis (RRP) is a rare disease of the aerodigestive tract caused by the Human Papilloma Virus (HPV) that manifests as profoundly altered phonatory and upper respiratory anatomy. Current therapies are primarily symptomatic; enhanced insight regarding disease-specific biology of RRP is critical to improved therapeutics for this challenging population. Multiplex PCR was performed on oral rinses collected from twenty-three patients with adult-onset RRP every three months for one year. Twenty-two (95.6%) subjects had an initial HPV positive oral rinse. Of those subjects, 77.2% had an additional positive oral rinse over 12 months. A subset of rinses were then compared to tissue samples in the same patient employing HPViewer to determine HPV subtype concordance. Multiple HPV copies (60–787 per human cell) were detected in RRP tissue in each patient, but a single dominant HPV was found in individual samples. These data confirm persistent oral HPV infection in the majority of patients with RRP. In addition, three novel HPV6 isolates were found and identical HPV strains, at very low levels, were identified in oral rinses in two patients suggesting potential HPV subtype concordance. Finally, somatic heteroplasmic mtDNA mutations were observed in RRP tissue with 1.8 mutations per sample and two nonsynonymous variants. These data provide foundational insight into both the underlying pathophysiology of RRP, but also potential targets for intervention in this challenging patient cohort.

## Introduction

Recurrent Respiratory Papillomatosis (RRP) is a rare disease characterized by recurrent papillomatous lesions of the upper aerodigestive tract in adults and children caused by the Human Papilloma Virus (HPV). Current therapies are limited beyond symptomatic, operative management potentially related to our collective lack of understanding regarding disease-specific biology of HPV in RRP in spite of increased insight regarding the role of HPV in the pathogenesis of malignancy. Oral HPV infection has been strongly associated with oropharyngeal squamous cell carcinoma^1^ and the natural history of oral HPV persistence has been evaluated in healthy individuals^2^. Our laboratory previously reported a much higher rate of oral HPV infection in patients with RRP compared to the general population^3^. Specifically, greater than 90% of patients with RRP lesions were HPV positive in the oral cavity. Based on these preliminary findings, we hypothesized that HPV DNA was likely released from RRP lesions into the oral cavity, and as such, patients with RRP maintain HPV positivity in the oral cavity beyond the reported clearance interval of approximately 7 months in healthy adults^2^.

The HPV subtype(s) identified in RRP lesions and oral DNA must be consistent to further this hypothesis. In the current study, we sought to provide foundational insight into these issues employing novel sequencing technology. Significant advances in genome sequencing allow for disease-specific HPV genomic data and metagenomic sequencing of human tissues allow for expanded and enhanced HPV detection methods for all HPV subtypes (182 types) as HPV sequences can be identified across the whole genome rather than just L1^4,5^. Specifically, metagenomics allows for characterization of the entire HPV genome and interactions between the HPV and host genomes^6^. In the current study, we employed a novel method of HPV detection using metagenomic data to analyze specimens containing HPV DNA isolated from oral rinse and laryngeal tissue samples from patients with RRP. We also sought to provide additional insight regarding potential mechanisms underlying RRP persistence/recurrence. In that regard, the role of aberrant mitochondrial function in cancer has been of particular interest for decades^7^. Specifically, mitochondrial DNA (mtDNA) mutations have been reported in renal adenocarcinoma, colon cancer cells, head and neck tumors, astrocytic tumors, thyroid tumors, breast tumors, ovarian tumors, prostate and bladder cancer, neuroblastomas, and oncocytomas^8–14^. mtDNA mutations have been considered essential factors for tumorigenicity^15–17^. Reactive oxygen species generated from mitochondria intensify the tumorigenic phenotype and induce additional genome mutations^18^. To date, mtDNA mutations have not been described in RRP and could potentially provide additional insight regarding the pathophysiology of RRP with the ultimate goal of identifying novel therapeutics.

## Results

### Oral HPV DNA persisted in patients with RRP

**Figure 1:**
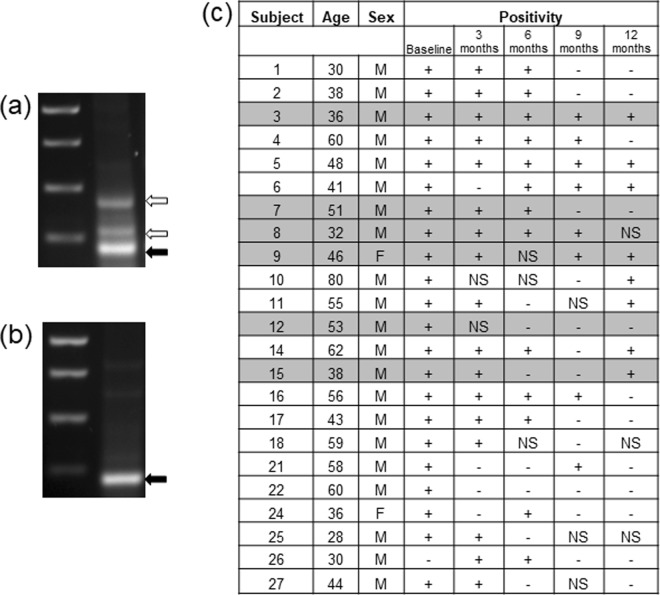
Analysis of oral rinse data. Representative gels of HPV positive (**a**) and negative (**b**) gels; solid arrow is GAPDH, open arrow is HPV positivity. Oral rinses were collected every 90 days over the course of 12 months (**c**); +=HPV positive, −=HPV negative, NS = missing sample or insufficient genetic content to complete analysis. Shaded subjects were also subjected to tissue analysis (subject numbers are not sequential). The complete gels from which (a) and (b) were obtained are included in the Supplemental File.

Twenty-three patients had multiple oral rinse specimens for analysis with a maximum of five specimens per subject; positivity was determined qualitatively via consensus across investigators (Fig. 1a,b). Twenty two (95.6%) subjects had an initial oral rinse specimen positive for HPV. Of those subjects, 77.2% had an additional positive specimen in at least one of samples collected 6–12 months following initial rinse acquisition. After the initial positive rinse, diverse patterns of HPV positivity were observed across subjects. Sixteen (59%) subjects had two consecutive positive specimens and 10 (37%) subjects had three consecutive positive specimens. Three (13%) subjects were HPV positive at all five time points (i.e., HPV positivity maintained for the 12-month study interval). Many subjects were initially positive for HPV, but then had an interval without positivity, only to be positive again at some point during the study (Fig. 1c). Across all subjects, including those with intermittent positivity, the average interval between the first and last positive oral rinse specimen was 320 days. Eight of the 23 subjects had at least one missing specimen during the study period.

### Single dominant HPV genome polymorphisms were found in rinses and tissue samples

**Figure 2:**
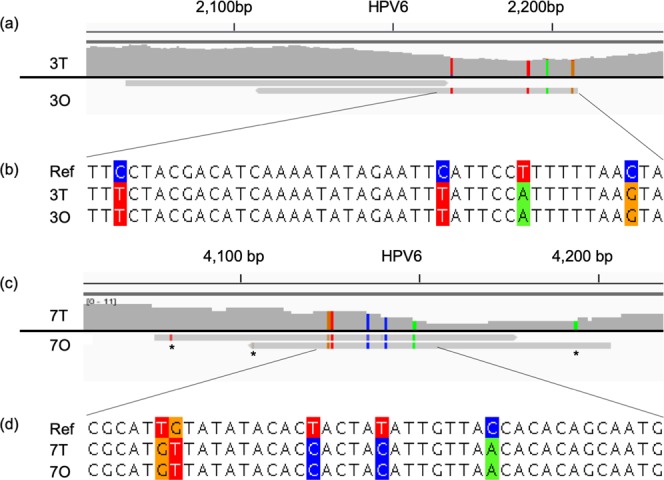
Alignments of 3T-3O and 7T-7O. The two reads from oral rinse samples shared the same SNP pattern with their matched specimens’ HPV genome. (**a**,**c**) are two IGV screenshots from 3T-3O and 7T-7O. In the 3T and 7T tracks, it showed HPV genome polymorphisms from tumor specimens with high abundant coverage; and in the 3O and 7O tracks, it showed two HPV reads from their oral rinse samples matched the same polymorphisms with their tumor specimens. (**b**,**d**) were multiple alignments of overlap regions in HPV genome among reference genome (Ref), tumor specimens and oral rinse. Asterisks in (**d**) indicated that the positions had low quality base pairs where the quality score was smaller than 20. The polymorphisms were colored.

Shotgun sequencing of both oral rinse and tissue samples was performed on a subset of patients who underwent operative resection of RRP lesions and oral rinse acquisition within a reasonable interval, as described in the Methods. HPV6 was detected in four RRP lesions and HPV11 was detected in the remaining two lesions. Copy number of HPV genomes in the lesion specimens ranged from 60–787 per human cell. According to the alignments of HPV reads to their prototype genomes, HPV in RRP specimens were different with the prototype HPV in genome level and a single dominant HPV strain in each individual sample. HPV reads found by sequencing rinses from two patients were identical to HPV sequenced from RRP tissue from those same patients (Fig. 2). Only two reads of HPV6 were identified in two oral rinse samples. Inspection of these sequences revealed identical polymorphisms, compared to the reference strain, with a larger number of reads in tissue samples.

### Three novel strains of HPV6 genomes from RRP specimens were reconstructed

**Figure 3:**
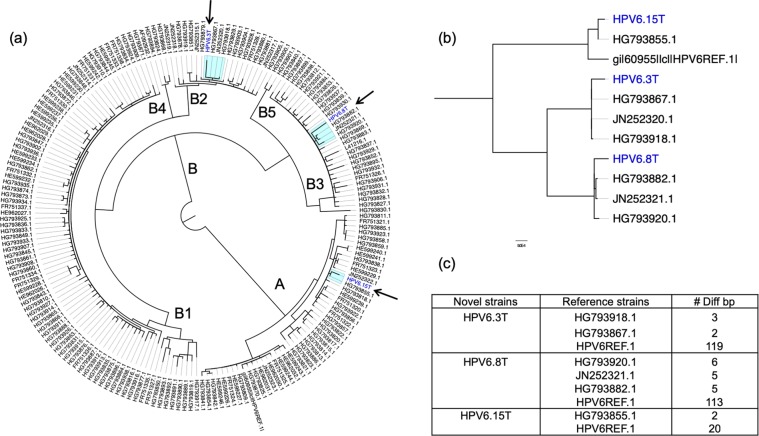
Phylogenetic analysis of three novel HPV isolates. (**a**) Maximum likelihood tree of global HPV6 genomes. It was inferred from the global alignment of the 193 complete HPV6 genome sequences including three novel genomes found in this study, one reference genome from PaVE and 189 genomes from GenBank. The Maximum likelihood tree has two lineages, A and B. Lineage B can be divided into five sub-lineages. Arrows point to HPV6 isolates. (**b**) The enlarged subtree from the complete maximum likelihood tree; three branches that contain the HPV6 isolates and their closest neighbors and prototype HPV6 are shown. (**c**) Summary of number of different nucleic acids between the HPV6 isolates and their neighbors and prototype HPV6. All three isolates were at least two base pairs different from the closest strains.

After alignment of HPV reads to HPV Reference genomes with Bowtie2, the genome of dominant HPV type was reconstructed, if all base pairs of the genome were covered by metagenomic reads more than 10 times. The genomes of three different HPV6 strains and one HPV11 strain were rebuilt from the four RRP tissue samples (3T, 8T, 9T and 15T) with at least 273 copies of HPV genome per human cell (Table 1). These four isolates had 20, 113, 119 and 24 base variations from the reference genome. To confirm whether those four HPV genomes were new, we built a maximum likelihood (ML) phylogenetic tree and Bayesian inference analyses for these three reconstructed HPV6 isolates with all 193 of the complete genomes for HPV6 and one reconstructed HPV11 with all 79 of the complete genomes for HPV11 (Fig. 3a,b). The phylogenetic tree indicated that three HPV6 strains came from different lineages (A, B2, and B3) and differed by at least two bases from any previously reported sequences (Fig. 3c); the reconstructed HPV11 strain was identical to HE962366.

**Table 1:** Summary of metagenomic analysis of tissue and oral rinse samples.

	Subject	^#^Total Reads	^#^Human Reads	Human autosome coverage	HPV type	HPV genome coverage	^#^HPV reads	HPV copy number	Multiplex HPV PCR
3	Tissue	7,610,476	6,490,818	0.21	HPV6	28.97	2257	269.89	+
	Rinse	80,467,174	37,687,186	1.25	HPV6, 51	0.03, 0.08	2, 6	0.05, 0.13	+
7	Tissue	5,266,202	4,095,050	0.14	HPV6	3.8	296	56.09	+
	Rinse	50,383,826	39,449,990	1.31	HPV6	0.03	2	0.05	+
8	Tissue	2,926,186	2,312,128	0.08	HPV6	12.99	1012	339.11	+
	Rinse	28,566,240	17,740,518	0.59	NA	0	0	0	+
9	Tissue	5,927,512	4,517,592	0.15	HPV11	47.56	3669	646.44	+
	Rinse	56,218,808	46,665,004	1.53	NA	0	0	0	+
12	Tissue	4,038,382	3,143,442	0.10	HPV11	2.5	193	48.20	+
	Rinse	52,063,294	38,071,086	1.26	NA	0	0	0	+
15	Tissue	2,543,838	1,843,244	0.06	HPV6	13.14	1024	432.37	+
	Rinse	91,833,436	66,618,328	2.22	NA	0	0	0	+

### Two nonsynonymous papillomatosis-related somatic mtDNA mutations were found in RRP tissue

mtDNA mutations were observed in the tissue samples and were identified as nucleotide changes in mtDNA compared to mtDNA in matched oral rinse samples to avoid the germline mutations. Somatic mtDNA mutations were observed in six RRP tissue specimens with 1.8 mutations per sample; all were heteroplasmic (allelic ratio from 10.8% to 24.2%; Table 2). Three of nine total somatic mtDNA mutations were in coding regions, one synonymous and two nonsynonymous mutations. Both nonsynonymous mutations, A336T in cytochrome c oxidase I (COI) and G215D in NADH ubiquinone oxidoreductase core subunit 5 (ND5), have not been reported previously in MITOMAP (https://www.mitomap.org/MITOMAP).

**Table 2:** Summary of somatic mtDNA mutations in RRP tissue.

Subject	Nucleotide position	Normal	Mutation	Allelic frequency	Amino acid change	mtDNA region
3T	414	T	G	0.147	—	D-loop
	2695	G	A	0.167	—	RNR2
	6909	G	A	0.119	A336T	COI
7T	8512	A	G	0.111	K49K	ATP8
	12980	G	A	0.242	G215D	ND5
8T	1316	T	C	0.133	—	RNR1
9T	125	T	G	0.108	—	D-loop
12T	15939	C	CT	0.136	—	tRNA-Thr
15T	622	G	A	0.222	—	tRNA-Phe

## Discussion

The pathogenesis and natural history of laryngeal HPV infection and the subsequent development of RRP is unknown. Previous theories include the concept of a localized deficiency in the immune response to the HPV infection^19^. In the current study, persistent oral HPV DNA was identified, and in the small subset of patients analyzed, homology was observed between the HPV subtype identified in the oral rinse and RRP tissue specimens confirmed via novel genomic techniques. In addition, significant mitochondrial mutations were identified in RRP lesions.

Consistent with our previous work, oral HPV infection was present in the vast majority of subjects with RRP. Longitudinal data acquisition was qualitative, and as such, the distinction between persistence of previously acquired infection or novel HPV infection with a new genotype was not determined. However, the subset of patients who underwent both rinse and laryngeal sample testing provided significant additional insight. In two of the six subjects, concordance between genotype in the oral rinse and laryngeal specimens was observed. This finding provided evidence to support two potential hypotheses which certainly warrant further investigation; a subset of RRP patients may be globally colonized in the upper respiratory tract and/or HPV DNA is released from RRP lesions to the oral cavity. However, most of the rinse specimens tested via shotgun sequencing did not have HPV 6/11 DNA despite high sensitivity of the methods employed. This finding indicates that HPV was very low in the oral cavity compared to RRP lesions; this decreased HPV yield may be related to DNA purification. D’Souza *et al*. reported that DNA purification markedly affected detection of HPV genomic DNA from oral rinse samples^20^. Depletion of human DNA prior to sequencing could potentially increase the sensitivity of detecting sparse HPV in the oral cavity by shotgun sequencing, which was not performed in this study^21^.

HPV alignment of RRP tissue demonstrated that the HPV genotypes had a stable genome. In other virus-host relationships, especially in RNA viruses, the virus itself is constantly mutating to meet the external pressures of host immunity. However, in the case of HPV, little genomic variability was observed, indicating that host-susceptibility is likely key in disease acquisition. Lucs *et al*. proposed that this lack of host reaction in RRP patients manifests as a local immune deficiency in upper respiratory tract tissues^22^. It was hypothesized that this deficiency may be due to overexpression of cyclooxygenase-2/Rac-1 pathway, resulting in an altered local inflammatory response. These data further strengthen the concept that patients with RRP have a diminished immune response to HPV within the upper respiratory tract.

To date, mitochondrial phenomena have not been implicated in RRP acquisition and disease progression. Lai *et al*. reported that HPV18 E2 protein colocalized with mitochondrial membranes and nearly 12% of proteins interacting with HPV18 E6 came from mitochondria; some participate in oxidative phosphorylation^23^. HPV16 E1^E4 was observed to bind mitochondria and lead to detachment of mitochondria from microtubules^24^. Furthermore, the E6 protein from HPV5 or HPV18 prevented release apoptosis inducing pro-apoptotic factor and cytochrome-c from mitochondria in UV-damaged epidermal cells^25^. Mitochondrial C150T polymorphism was reported to be related to increased risk of cervical cancer and HPV infection^26^. Clinically, the risk of HPV related cervical cancer is increased in patients with Amerindian mtDNA haplo-group B2^27^. Although no direct inferences between mitochondrial mutations and RRP can be asserted at this time, these data serve as a foundation for future investigation.

Since both nonsynonymous mutations, A336T in COI and G215D in ND5, have not been reported previously, we hypothesize that healthy subjects will not present with these mutations; however, further investigation is certainly warranted. In addition, the connection between RRP pathophysiology and these mutations remains unclear. However, at position 215 of ND5, Glycine (G) is conserved across multiple species: *balaenoptera musculus*, *bos taurus*, *mus musculus*, *phoca vitulina*, *rattus norvegicus*. This conservation may lead to dysfunction of ND5. Furthermore, at position 336 of COI, either Alanine (A) or Proline (P) is conserved across multiple species. Of note, both of these amino acids have nonpolar side chains, but the mutated T has a polar side chain; this COI mutation may also lead to dysfunction. These hypotheses warrant further investigation.

The current study is not without limitations. As with previous studies employing qualitative PCR, the longitudinal data are limited by diminished capacity to capture multiple concurrent HPV subtypes^3^. In addition, the current study did not include analysis of control rinses; our data in patients with RRP were qualitatively compared to impressive, previously-published results regarding the prevalence of oral HPV infection in the United States^28^. Conversely, the sensitivity of shotgun sequencing analysis for HPV DNA was limited by the overabundance of human DNA in our specimens. Although these two methods are both useful, each carries non-trivial limitations with regard to data yield. Additionally, the technique is prone to false-negatives due to saturation by competing HPV DNA in small amounts^29^. Regardless, these data provide insightful and foundational information regarding this particularly morbid disease process.

## Methods

### Subjects

The current study was approved by the Institutional Review Board at the New York University School of Medicine; informed consent was obtained from all participants. In addition, all methods in the current study were performed in accordance with all relevant guidelines and regulations. Oral rinses were collected from 23 subjects with pathology confirmed adult-onset RRP (21 male, 2 female) every 90 days over 12 months. Subjects ranged from 30 to 80 years with a mean age of 49 years (SD = 13 years). Tissue blocks were analyzed from a subset of six of subjects (5 male, 1 female; mean = 43 years, SD = 8 years) based on close temporal proximity between the acquisition of both oral rinse and tissue samples during operative resection of disease as a component of standard clinical care. The interval between oral rinse and tissue acquisition ranged from 0 to 18 weeks with a mean of 5.7 weeks.

### Oral rinse and DNA extraction

Subjects were instructed to swish 5 mL of Scope® mouthwash without gargling for one minute. Samples were then sealed and stored for no more than one week at 4 °C prior to DNA extraction. For DNA extraction, samples were spun for 10 minutes at 3200 *g*. DNA in the cell-free supernatant was precipitated with the isopropanol/glycogen solution and pelleted for 10 minutes at 2000 g, as previously described^3^. The pellet was resuspended with 200 µL DNA Hydration Solution (Qiagen) and DNA was extracted using the Gentra Pure-gene Buccal Cell Kit (Qiagen), according to the manufacturers’ protocol, modified slightly. Rinse samples were spun for 10 minutes at 3,200 g and after transferring the supernatant to a isopropanol/glycogen solution, samples were mixed by inverting 50 times and spun again for 10 minutes at 2,000 g. DNA hydration solution (200 mL) was added to the pellet.

### Multiplex polymerase chain reaction (PCR) for HPV

PCR was performed using the biotinylated PGMGY09/11 consensus primer system (Invitrogen, Grand Island, NY) to detect a broad spectrum of HPV genotypes. Briefly, 10pmols of the PGMY09/11 and 2.5pmols of the PCO4/GH2O sequence were employed per reaction. For each reaction, 4 mM MgCl2, 400 µM dNTP, 2.5 mL of GC enhancer, and 7.5U of Amplitaq Gold DNA Polymerase were used per 50 mL reaction. Subsequently, 8 µL of DNA plus water were used per reaction with RNAse-free water as a loading control. PCR was performed using an Eppendorf Mastercycler (Hauppauge, NY) with the following settings: 50 °C for 2 minutes, 95 °C for 10 minutes and 40 cycles of 95 °C for 1 minute, 55 °C for 1 minute, and 72 °C for 1 minute. This protocol was followed by a final extension at 72 °C for 5 minutes and then 4 °C indefinitely. PCR products were then analyzed on a 2% agarose gel; distinct bands and smears were considered positive results for HPV DNA if clear expression of β-actin was observed.

### Tissue acquisition and DNA extraction

All RRP tissue specimens were obtained during intraoperative, endoscopic resection and were received as formalin-fixed paraffin blocks. The diagnosis of RRP was made by standard histopathological examination. Blocks were sectioned at 20 µm and total genomic DNA was extracted using BiOstic FFPE Tissue DNA Isolation Kit (Mo Bio Carlsbad, CA).

### Metagenomic sequencing and alignments

Paired-end metagenomic sequencing was performed to determine concordance of HPV subtype between RRP specimens and oral rinse employing the Illumina HiSeq 2500 platform (read length, 100 bp) with 100 ng template DNA per sample and 12 samples per lane. Sequencing reads were trimmed by TRIMMOMATIC^30^ and the number of high quality reads per sample was from 2,926,186 to 91,833,436. All high-quality reads were aligned to the human genome (GRCh38) to obtain average coverage across all 22 autosomal chromosomes. A HPV profile with HPV coverage for each sample was generated by HPViewer. With two copies of human autosomal DNA per cell, copy number of HPV per human cell was defined as:

1 $${\rm{HPV}}\,{\rm{copy}}\,{\rm{number}}\,{\rm{per}}\,{\rm{cell}}=\frac{{\rm{HPV}}\,{\rm{average}}\,{\rm{coverage}}}{{\rm{autosomome}}\,{\rm{DNA}}\,{\rm{average}}\,{\rm{coverage}}}\times 2$$

### HPV genome reconstruction from metagenomic data

Only four specimens had sufficient sequencing depth to reconstruct the local HPV genome. The entire HPV genome was covered over 10 times by short DNA reads from the four samples; the HPV genome covered by the reads from the other two samples were lower than five, limiting the ability to reconstruct the local genome. Thus, shotgun sequencing data from three HPV6-positive specimen samples were aligned to the entire HPV6 reference genome and the data from the HPV11-positive sample was aligned to the entire HPV11 reference genome via Bowtie2^31^. SAMTools/1.3^32^, Picard/2.6.0, and GATK/3.6^33^ were then applied to de-duplicate and re-align insertion/deletions and to recalibrate base quality score. Single nucleotide polymorphisms (SNP) and small insertions and deletions (INDEL) were identified by GATK HaplotypeCaller. Based on SNP and INDEL information, complete genomes were constructed for those four isolates, three strains of HPV6, and one strain of HPV11.

### Phylogenetic tree construction for HPV6 and HPV11

Four HPV genomes in the RRP lesions were constructed. The HPV6 phylogenetic tree was built from 193 HPV6 complete genomes including novel genomes identified in this study, one reference genome from PaVE, and 189 genomes from GenBank^34^. GenBank accessions of those 189 genomes are HG793809 to HG793938 and FR751320 to FR751338, HE599226 to HE599246 and HE962026 to HE962032, and JN252314 to JN252323 and non-prototypic HPV6a (accession no. L41216) and HPV6vc (accession no. AF092932) genomes. Because of the circular structure of HPV, the genomes were linearized at the first ATG of the E6 ORF. The HPV11 phylogenetic tree was built from 79 HPV11 complete genomes including one genome found in this study, one reference genome from PaVE, and 77 genomes from GenBank. GenBank accession of those 77 genomes are LN833161 to LN833190 and EU918768, FR872717, N870021, FN870022, and FN907957 to FN907964, JQ773408 to JQ773412, HE574701 to HE574705, JN644141 and HE611258 to HE611274, HE962023 to HE962025, and HE962365 to HE962368. All genomes were linearized at the first ATG of the E6 ORF.

All HPV6 genomes and HPV11 genomes were aligned using MUSCLE3.8.31^35^, respectively. RAxML 8.2.9 was employed to build maximum likelihood trees under a GTRCAT substitution model with 1,000 bootstrapping replicates^36^. MrBayes-3.2.6 was employed to construct Bayesian trees with 10,000,000 generations for Markov Chain Monte Carlo runs under a General Time Reversible model (GTR) with gamma-shaped distribution rate variation and a proportion of invariable sites^37^. Phylogenetic trees with a midpoint rooted were visualized by FigTree v1.4.3^38^. MEGA7 was used to compare the number of DNA difference between close strains^39^.

### mtDNA somat1ic SNP calling

All high-quality reads were aligned to the human mitochondrial genome (NC_012920) by Burrows-Wheeler Aligner (BWA/0.7.7 bwa-mem)^40^. SAMTools/1.3, Picard/2.6.0, and GATK/3.6 were then applied to de-duplicate and re-align insertion/deletions and to recalibrate base quality score. MuTect2 was used to identify somatic mtDNA mutations from RRP tissue compared to oral rinse mtDNA to eliminate germline SNP. Annotation of SNPs was conducted by ANNOVAR (version 2017Jul16)^41^.

## Supplementary information

Supplementary Info-Figure 1
