# Supplementary material for: Mitochondrial somatic mutations and the lack of viral genomic variation in recurrent respiratory papillomatosis
Source: Sci Rep. 2019 Nov 12;9:16625. doi: 10.1038/s41598-019-53148-8 (PMC6851396; doi:10.1038/s41598-019-53148-8)
Supplement: Supplementary file 1 — Supplementary Info-Figure 1 [file 41598_2019_53148_MOESM1_ESM.docx]

**Mitochondrial somatic mutations and the lack of viral genomic variation in recurrent respiratory papillomatosis**

Yuhan Hao, MS^1^

Ryan Ruiz, MD^2^

Liying Yang, MD^3,4^

Antonio Galvao Neto, MD^3^

Milan R. Amin, MD^2^

Dervla Kelly, PhD^3^

Stratos Achlatis, MD^2N^

Scott Roof, MD^2^

Renjie Bing, MD^2^

Kasthuri Kannan, PhD^3,5^

Stuart M. Brown, PhD^5^

Zhiheng Pei, MD, PhD^3,4,6^

Ryan C. Branski, PhD^2^

^1^Center for Genomics and Systems Biology, Department of Biology, New York University, New York, NY

^2^Otolaryngology-Head and Neck Surgery, New York University School of Medicine, New York, NY

^3^Department of Pathology, New York University School of Medicine, New York, NY

^4^Department of Medicine, New York University School of Medicine, New York, NY

^5^Applied Bioinformatics Laboratories, New York University School of Medicine, New York, NY

^6^Department of Veterans Affairs New York Harbor Healthcare System, New York, NY

Support for this work was provided by the National Institute of Dental and Craniofacial Research, National Institute of Allergy and Infectious Diseases, and National Cancer Institute of the National Institutes of Health (R21DE025352, R01AI110372, R01CA204113, and U01CA182370) as well as the American Society of Pediatric Otolaryngology Dustin Micah Harper Recurrent Respiratory Papillomatosis Research Grant.

ZP is staff physician at the Department of Veterans Affairs New York Harbor Healthcare System. The content is the sole responsibility of the authors and does not necessarily represent the official views of the National Institutes of Health, the U.S. Department of Veterans Affairs or the United States Government.

The authors have no conflicts of interest or financial disclosures relevant to the data contained in this manuscript.

**Address all correspondence to**:

Ryan C. Branski, Ph.D.

Otolaryngology-Head and Neck Surgery

New York University School of Medicine

345 East 37th Street, Suite 306

New York, NY 10016

Phone: (646) 754-1207

[ryan.branski@nyumc.org](mailto:ryan.branski@nyumc.org)

**Supplemental Information**

Original gels from which components of Figure 1 were obtained.

**
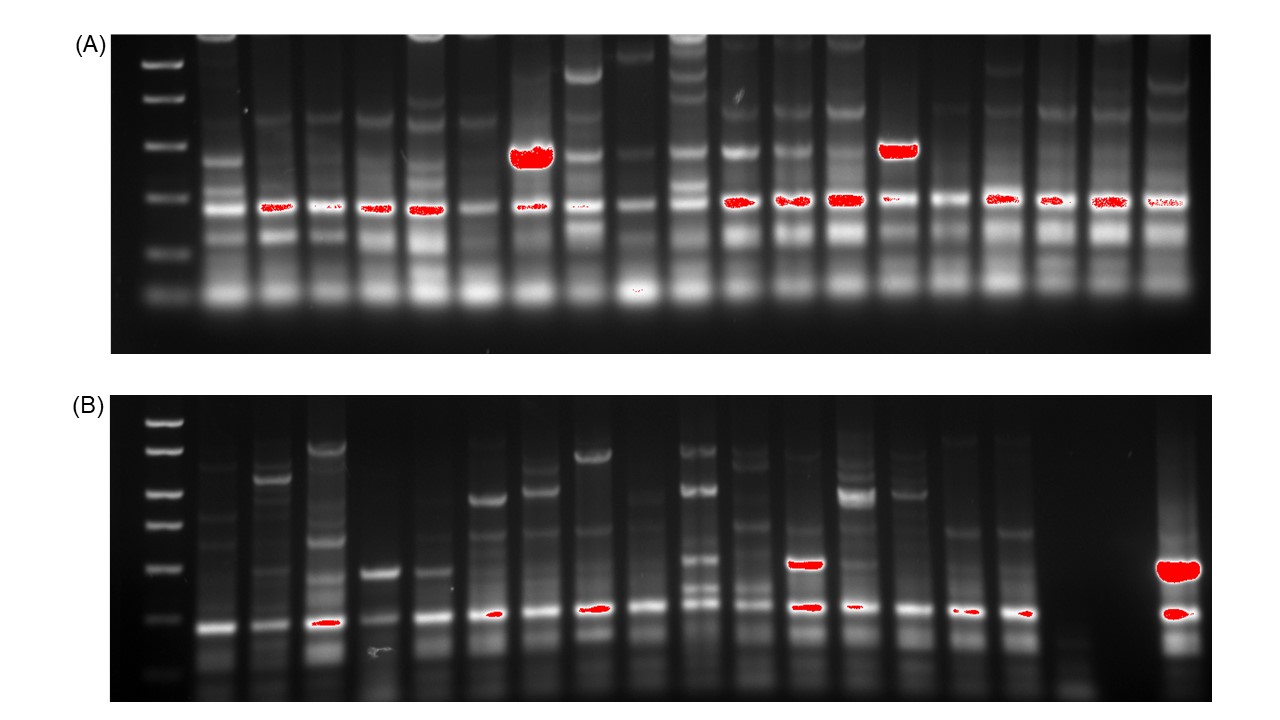
**
